# Structural features of isomerizable aspartyl residues in human α-crystallins

**Published:** 2012-07-04

**Authors:** Ken-ichi Shimizu, Akiko Kita, Noriko Fujii, Kunio Miki

**Affiliations:** 1 Research Reactor Institute, Kyoto University, Kumatori, Sennan, Osaka, Japan; 2 Graduate School of Science, Kyoto University, Sakyo-ku, Kyoto, Japan

## Abstract

**Purpose:**

The aspartyl (Asp) residues 58 and 151 in αA-crystallin, and Asp36 and Asp62 in αB-crystallin in human lenses are known to be highly isomerized with aging. We investigate structural environments of these isomerizable aspartyl residues in α-crystallins of human lenses.

**Methods:**

To perform limited proteolysis experiments of purified human αA- and αB-crystallins, endoproteinase Asp-N (EC 3.4.24.33), which selectively cleaves the peptide bonds at the amino side of aspartyl and cysteic acid residues, was employed. By proteolysis approach coupled with the time-of-flight mass spectrometry (TOF-MS) method, we determined the cleavage points along protein sequences.

**Results:**

Proteolysis by endoproteinase Asp-N occurred preferentially at the site of isomerizable aspartyl residues in αA- and αB-crystallins.

**Conclusions:**

It is found that isomerizable aspartyl residues in α-crystallins in human lenses were located not only in the solvent accessible area but also at regions displaying inherent conformational flexibility.

## Introduction

**Figure 1:**
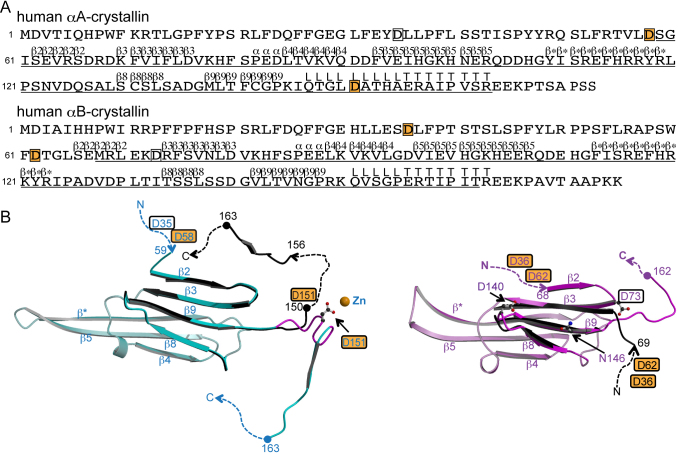
The structures of αA- and αB-crystallins. **A**: Primary and secondary structures of αA- and αB-crystallins [13]. The secondary structure is denoted by upper letters; β, α, L and T indicate strands, helices, loops and COOH-terminal tail, respectively. Regions for which structural information is available in bovine αA-crystallin (PDB ID: 3L1E) and human αB-crystallin (PDB ID: 3L1G) are underlined [13]. The letters in boxes indicate aspartyl residues in which digestion by Asp-N occurs, and those on an orange background indicate isomerizable residues in human αA- and αB-crystallins. **B**: Tertiary structures of αA- (left) and αB- (right) crystallins. Numbers indicate the amino acid positions. The dashed lines indicate disordered regions. The letters in boxes indicate aspartyl residues in which digestion by Asp-N occurs, and those on an orange background indicate isomerizable residues in αA-crystallin and in αB-crystallin in vivo. Asp151 in αA-crystallin, and Asp73, Asp140 and Asn146 in αB-crystallin noted in the text are drawn in ball-and-stick models to make clear their positions. The figures were drawn by MOLSCRIPT [23] and RASTER3D [24]. Left: The recombinant bovine αA-crystallins (cyan, PDB ID: 3L1E). The β strands 2, 3, 8 and 9, the hinge loop region noted in the text (Gln147 - Ala155) and the C-terminal tail of the other bovine αA-crystallin crystal structure (black, PDB ID: 3L1F) are superposed [13]. The hinge loop region is colored in magenta in 3L1E. Right: One of the crystal structures of recombinant human αB-crystallins (pink, PDB ID: 3L1G). The β strands 3, 8 and 9 and available NH_2_-terminal region of the representative model in NMR structure (black, molecule A in model 1 in PDB ID: 2KLR) are superposed [13,15].

The mammalian eye lens is composed of three major structural proteins, α-, β- and γ-crystallins [1]. α-Crystallin, with a molecular mass of approximately 800 kDa, is a globular protein comprised of two kinds of polypeptides, αA-crystallin and αB-crystallin, that share 57% sequence homology (Figure 1A) [2]. With aging, the crystallins undergo various modifications with subsequent aggregation and insolubilization [3]. Aggregation and insolubilization are related to post-translational modifications such as racemization and isomerization [4-6]. αA- and αB-crystallins are small heat-shock proteins that protect other proteins from aggregation and play central roles in maintaining lens transparency and refractive properties [7].

The aspartyl (Asp) residues 58 and 151 in human αA-crystallin and Asp36 and Asp62 in human αB-crystallin from elderly donors are highly inverted from the L-isomer to the D-isomer, and they are isomerized from the normal α linkage to the β linkage of the peptide-bond [5,6]. The oligomerization patterns of α-crystallins are still unclear. However, the structural environments of these “isomerizable” residues in the recombinant proteins are supposed to be similar to those of the intact proteins from elderly donors, since the previous kinetic studies of αA-crystallin indicated that isomerization of Asp58 and Asp151 occurs also in the recombinant human αA-crystallin [8]. The protein folding can be affected by D-isomerization of aspartyl residues, and the β linkage of aspartyl residues affects the quaternary structure because of conformational changes of the main chain. The size and size distribution of the α-crystallin assembly are related to the number of isomerized aspartyl residues, and the self-association of α-crystallin is correlated with changes in chaperone activity [3]. As a result, α-crystallin containing large amounts of D-β-Asp undergoes abnormal aggregation to form large and heterogeneous assemblies, which decrease its chaperone activity. Such a decrease of chaperone activity causes abnormal aggregation or insolubilization of other lenticular proteins, which is associated with diseases such as cataract formation.

It has been reported that the isomerization of Asp/Asn residues in various peptides occurs via a succinimide intermediate [9-11]. Succinimide formation undergoes rapidly when the carboxyl side of Asp/Asn is a small residue, such as glycine, alanine, or serine, because such residues have little steric hindrance. The isomerization is affected not only by the sizes of neighboring residues but also by the environments around Asp/Asn in the protein structures. A previous study showed that the racemization rates of Asp58 and Asp151 in the recombinant human αA-crystallin were twice higher than those of the model peptides [8], and that racemization at the possible sites of Ser139-Asp140-Gly141 or Val145-Asn146-Gly147 in αB-crystallin was not observed [6]. Therefore, structural information on α-crystallins would contribute to understanding of the molecular mechanisms of the isomerization of aspartyl residues.

Recently, the crystal structures of three truncated forms of human αB-crystallins were determined (Figure 1B) [12-14]. These three truncates do not contain isomerizable aspartyl residues. The nuclear magnetic resonance (NMR) and small-angle X-ray scattering (SAXS) structures of whole human αB-crystallin are available [15], but those isomerizable aspartyl residues are invisible in the solution structure. On the other hand, the crystal structures of truncated αA-crystallin from bovine and zebrafish have been determined (Figure 1B) [13,16]. These truncated proteins contain an aspartyl residue corresponding to Asp151 of human αA-crystallin that could be isomerized; this aspartyl residue is available in these crystal structures. However, other isomerizable aspartyl residues (Asp58 in αA-crystallin, and Asp36 and Asp62 in αB-crystallin) do not exist in these truncated proteins, and thus their structural environments are unknown.

Limited proteolysis has been used as a probe to gain insights into folding for proteins of unknown structure [17]. It has been reported for bovine lens α-crystallins and human αB-crystallin [18,19]. In these studies, several proteases such as trypsin, chymotrypsin, subtilisin, and thrombin were employed, but endoproteinase Asp-N (EC 3.4.24.33), which selectively cleaves peptide bonds at the amino side of aspartyl and cysteic acid residues, was not used.

We examined the limited proteolyses of the recombinant human αA- and αB-crystallins by using endoproteinase Asp-N to investigate the structural environments of aspartyl residues. Recombinant αA- and αB-crystallins have sedimentation coefficients 11–13S and 15S, respectively [3]. The results show that proteolysis by Asp-N occurs preferentially at aspartyl residues that could be isomerized among other aspartyl residues located at various positions of human αA- and αB-crystallins.

## Methods

### Protein expression and purification

αA-crystallin in the vector pET3d was expressed in the BL21(DE3)pLysS strain of *E. coli* [20]. The soluble fraction was applied to an ion exchange column (SuperQ-Toyopearl 650S; Tosoh, Minato-Ku, Tokyo, Japan) equilibrated with 20 mM tris(hydroxymethyl)aminomethane (Tris) buffer (pH 8.0). αA-crystallin was eluted with a linear NaCl gradient from 0 to 400 mM. The fractions containing αA-crystallin were pooled, concentrated by ultrafiltration (Amicon Ultra; Millipore, Billerica, MA), and filtrated through a 0.22-μm-pore-size filter (Millipore). Subsequently, the sample was loaded onto a gel filtration column (HiLoad 16/60 Superdex 200 prep grade; GE Healthcare, Uppsala, Sweden), which was equilibrated with 20 mM Tris buffer (pH 8.0) containing 150 mM NaCl. Finally, the fractions were dialyzed and applied onto an ion exchange column (Mono Q HR5/5; GE) equilibrated with 20 mM Tris buffer (pH 8.0). The fractions containing αA-crystallin were eluted with a linear gradient from 0 to 400 mM NaCl.

The αB-crystallin in the vector pET23d was expressed in the BL21(DE3)pLysS strain of *E. coli* [20]. The soluble fraction of αB-crystallin was applied to an ion exchange column and to a gel filtration column in the same manner as for αA-crystallin, but it was not applied onto the second ion exchange column (the last column) because the samples were sufficiently purified by the first columns that a single protein band was expected on SDS–PAGE.

### Proteolysis of α-crystallins

For the limited proteolysis experiments, 1.0 mg/ml αA- and αB-crystallin samples in 20 mM Tris buffer (pH 8.0) were prepared. For protease digestion, endoproteinase Asp-N (Roche, Basel, Switzerland) was used. Digestion was performed by addition of 1:10, 1:100, or 1:1,000 (w:w) ratio of protease reagent to α-crystallins to determine the best reaction conditions for elucidating the cleavage process. Digestion experiments were performed at room temperature. Digestion was quenched by adding SDS sample buffer or by adding a matrix solution of mass spectrometry at the time points of 10 min., 30 min., 1 h, 3 h, and 24 h. The digestion mixtures were confirmed by SDS–PAGE (data not shown), and analyzed by a more sensitive method, matrix-assisted laser desorption/ionization time of flight mass spectrometry (MALDI-TOF MS), as described below.

### Mass spectrometry

All mass spectra were collected on a Voyager-DE PRO Biospectrometry Workstation (Applied Biosystems, Foster City, CA) using the MALDI-TOF MS method. The data were collected in linear mode as a signal of positive ions. As a matrix, 10 mg/ml sinapinic acid dissolved in a solution containing 50% acetonitrile with 0.1% trifluoroacetic acid was used. The sample solution was added to an equal volume (0.5 μl) of the matrix on the plate, and then dried. Each sample was present at the level of a few pmol per spot. The data were smoothed using the default setting of Voyager Data Explorer software. Peptides were identified by the FindPept tool on the ExPASy Proteomic Server with 1,000 ppm tolerance.

## Results

### Digestion of αA-crystallin by Asp-N

**Figure 2:**
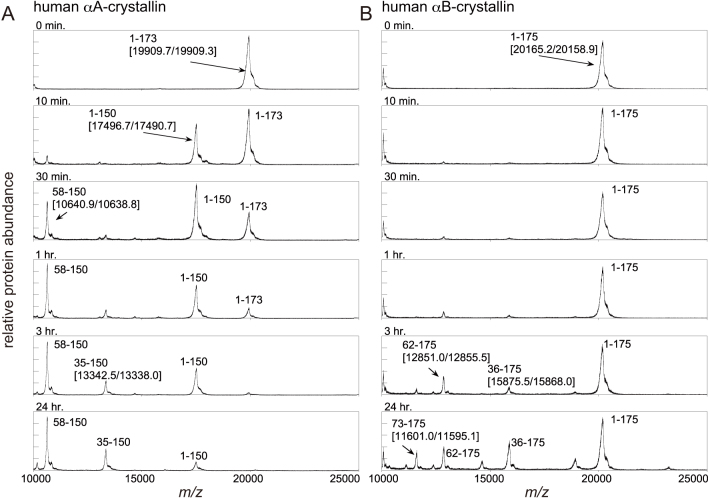
Mass spectra transformed to the mass scale of α-crystallins. The vertical and horizontal axes correspond to the relative protein abundance and *m/z*, respectively. The expected cleavage fragments and [measured mass values in MALDI-TOF spectra (Da)/theoretical value (Da)] are indicated. Spectra were acquired for each sample in which digestion by endoproteinase Asp-N was quenched at 0 min (not digested), 10 min, 30 min, 1 h, 3 h, and 24 h. **A**: Human αA-crystallin. **B**: Human αB-crystallin.

Digestion of αA-crystallin was performed by the addition of a 1:1,000 (w:w) ratio of endoproteinase Asp-N. The spectra of MALDI-TOF MS transformed to a mass scale of αA-crystallin are shown in Figure 2A. These results indicate that the cleavage of the peptide bond between the residues Leu150 and Asp151 dominated, and then the cleavage product of αA (Asp58-Leu150) appeared. Afterwards, the other cleavage product of αA (Asp35-Leu150) followed.

### Digestion of αB-crystallin by Asp-N

Digestion of αB-crystallin was also performed by the addition of a 1:1,000 (w:w) ratio of endoproteinase Asp-N. The spectra of MALDI-TOF MS transformed to a mass scale of αB-crystallin are shown in Figure 2B. In the MALDI-TOF MS spectra, two predominant peaks, corresponding to the cleavage products of αB (Asp36-Lys175) and αB (Asp62-Lys175), appeared after 1 h of incubation. The third peak, corresponding to the cleavage product of αB (Asp73-Lys175), appeared after 3 h of incubation.

## Discussion

It was previously reported that the limited proteolysis of a globular protein occurs not only at exposure/accessible area but also at regions which display inherent conformational flexibility, because limited proteolysis by proteases requires a conformational change of a polypeptide chain of the substrate protein [21]. The present results show that Asp-N predominantly degraded Asp151 in human αA-crystallin (containing 15 aspartyl residues) and Asp36, Asp62 and Asp73 in human αB-crystallin (containing 11 aspartyl residues). Asp35 and Asp58 were the subsequent proteolytic targets in αA-crystallin. A comparison of available αB-crystallin structures indicates that the NH_2_-terminal region (upstream from the β3 strand) adopts different conformations (Figure 1B: right) [13,15]. Asp73 in αB-crystallin is located at the point of bifurcation, that is, at the inherently flexible region between a mobile NH_2_-terminal region and the β3 strand. In the case of αA-crystallin, the crystal structures of bovine αA-crystallins show that the COOH-terminal region (following the β9 strand) takes different conformations in the presence and absence of zinc ion, respectively [13]. The residue corresponding to Asp151 in human αA-crystallin is located in the “hinge loop” region consisting of Gln147-Ala155 between a mobile COOH-terminal region and the β9 strand (Figure 1B: left) [13]. It is expected by their amino acid similarity that the whole conformations of bovine and human αA-crystallins are essentially similar, and therefore, Asp151 is thought to be located in the “hinge loop” region also in human αA-crystallin [13]. These results are consistent with a previous study showing that proteolyses of globular proteins occur in the inherently flexible region [21,22]. By the present proteolyses, it is expected that Asp36 and Asp62 in human αB-crystallin, and Asp35 and Asp58 (subsequent cleavage sites) in human αA-crystallin, are also located not only in the solvent accessible area but also in the inherently flexible region.

In previous reports, Asp58 and Asp151 in αA-crystallin and Asp36 and Asp62 in αB-crystallin from elderly human donors were shown to be highly isomerized [5,6]. As mentioned above, these isomerizable aspartyl residues are thought to be located in the inherently flexible regions. Therefore, it is concluded that the isomerization of aspartyl residues requires not only 1) to have the neighboring residues with a small side chain, but also 2) to be located in the inherently flexible region between domains. These two requirements could explain why residues Asp140 and Asn146 in human αB-crystallin, which have neighboring amino acid residues with a small side chain, could not be isomerized. The structure of human αB-crystallin indicates that Asp140 and Asn146 are not located between domains but in the loop region between β8 and β9 strands (Asp140) and in the β9 strand (Asn146), taking a rigid environment in the β8-β9-β3 anti-parallel β-sheet (Figure 1B). The three-dimensional structures of the human αA- and αB-crystallins, including the aspartyl residues in which proteolysis occurs, will provide more information and reveal whether these hypotheses are appropriate or not.
